# Decreased lung hyaluronan in a model of ARDS in the rat: Effect of an inhibitor of leukocyte elastase

**DOI:** 10.3109/03009734.2011.622812

**Published:** 2012-02-15

**Authors:** Chul Min Ahn, Håkan Sandler, Tom Saldeen

**Affiliations:** ^1^Department of Surgical Sciences, University of Uppsala, Uppsala, Sweden; ^2^Pulmonary Division, Department of Medicine, Kangnam Severance Hospital, Yonsei University College of Medicine, Seoul, Korea; ^3^Institute of Chest Diseases, Kangnam Severance Hospital, Yonsei University College of Medicine, Seoul, Korea

**Keywords:** Acute lung injury, acute respiratory distress syndrome, hyaluronan, leukocyte elastase inhibitor, pulmonary edema

## Abstract

**Background.:**

Hyaluronan (HA) is a component of the extracellular matrix in lung tissue and is normally present at low concentrations in blood. HA is rapidly cleared from blood by the liver. Increased concentrations of plasma HA have been found in patients with acute respiratory distress syndrome (ARDS). We investigated changes in HA levels in plasma, bronchoalveolar lavage fluid (BALF), and lung, and their relationship to pretreatment with a leukocyte elastase inhibitor in a rat model of ARDS.

**Methods.:**

Rats were randomly assigned to three groups: control, thrombin, and thrombin plus elastase inhibitor. By use of a radiometric assay, HA was measured in lungs, BALF, and plasma. Tissue samples from the lungs were stained for HA and examined microscopically. Liver circulation and cardiac output were monitored using radiolabeled microspheres.

**Results.:**

Infusion of thrombin produced a pronounced increase in wet weight to dry weight ratio, and relative lung water content. This increase was blunted by a leukocyte elastase inhibitor. A decrease in lung HA and increases in both BALF and plasma HA were found. The leukocyte elastase inhibitor counteracted not only the decrease in lung tissue HA, but also the increase in plasma HA. Histologically, there was decreased HA-staining of peribronchial and perivascular areas in the injured rat lung. Decreased liver perfusion was observed after infusion of thrombin.

**Conclusions.:**

The decrease in lung HA may be involved in the development of pulmonary edema in this ARDS model, and leukocyte elastase may be one cause of this decrease. In addition, an elevated plasma HA level may be an indicator of lung injury.

## Introduction

Hyaluronan (hyaluronic acid, HA) is an important component of the extracellular matrix and is widely distributed in the lung parenchyma in both man and rat. HA has been shown to produce distinct biological effects depending on the molecular weight (MW). HA exists predominantly in a high-MW form (HMW-HA; > 500 kDa) under physiological conditions (1). Per se or in conjunction with collagen fibers, proteoglycans, and other macromolecular elements of the extracellular matrix, HA provides cellular support and stabilization of structures and regulates cell–cell adhesion and the movement of interstitial fluid and macromolecules in the lung (2,3). In contrast, a low-molecular-weight HA (LMW-HA; < 500 kDa) can function as an intracellular signaling molecule in inflammation and has been found to be pro-inflammatory (4). The concentration of HA in the interstitium is the result of a dynamic process of equilibrium between biosynthesis and degradation, influencing serum and tissue HA levels. However, this balance is altered in many diseases. Increased deposition of HA in lung tissue has been observed in bleomycin-injured rats (5), in a rat model of monocrotaline-induced pulmonary hypertension (6), acute respiratory distress syndrome (ARDS) in man (7), premature monkeys (8), and oxygen-induced lung injury in rabbit pups (9), whereas increased HA synthesis has been reported in non-human primates with ARDS (10). A decrease in lung HA has been reported in group B streptococcal pneumonia in neonatal piglets (11) and was observed 10 days after monocrotaline-induced injury in rats, before the onset of pulmonary hypertension (6). HA was not elevated in lavaged lung tissue in radiation-induced lung disease in rats (12).

Experimental studies have demonstrated a link between the interstitial accumulation of HA and pulmonary edema in bleomycin-induced alveolar injury in the rat (5). Serum HA has been suggested as a prognostic factor in patients with ARDS (13). However, the mechanisms responsible for these HA abnormalities are poorly understood.

Thrombin-induced lung injury, an animal model of ARDS, is characterized by increased pulmonary vascular permeability to protein, leading to severe pulmonary edema (14). The pathophysiology of this injury may involve leukocytes (15) and leukocyte elastase (14). Reactive oxygen species and myeloperoxidase derived from stimulated polymorphonuclear leukocytes are able to degrade HA directly (16,17).

Leukocyte elastase from activated neutrophils may cleave extracellular matrix (ECM) components such as proteoglycans, collagen, and fibrin that are bound to HA (18). Thus, HA bound to the ECM may be released and subjected to oxygen-induced attacks. It is therefore possible that the lung injury in this model may lead to local degradation of HA.

We hypothesized that lung HA might be decreased in rats with thrombin-induced lung injury due to an increased wash-out of HA. Furthermore, we proposed that the lung injury might be accompanied by elevated concentrations of circulating HA and that this elevation might be partly due to a decreased uptake of HA in the liver, as a result of diminished liver perfusion.

We therefore measured concentrations of HA in lung tissue, bronchoalveolar lavage fluid (BALF), and plasma and studied lung tissue microscopically for the presence of HA in rats with thrombin-induced lung injury. We also investigated the effect of a synthetic human leukocyte elastase inhibitor on the levels of HA in thrombin-induced pulmonary edema. In addition, we studied cardiac output and liver circulation, since the liver plays an important role in HA degradation.

## Material and methods

### Animals

Eighty-nine male Sprague-Dawley rats (Alab, Stockholm, Sweden) were used in this study. Seventy-five of them were used for determination of HA and divided into three treatment groups: control (*n* = 24), thrombin (*n* = 40), and elastase inhibitor plus thrombin (*n* = 11). All specimens for HA determination used in this study were obtained from our previous studies. In separate experiments, cardiac output was measured at intervals in six rats, and liver blood flow was determined in eight other rats given thrombin; each rat being its own control. All rats weighed between 200 and 250 g and had free access to food (Ewos rat pellet) and tap water. The present study was approved by the ethics committee at Uppsala University.

### Materials

Bovine thrombin (Topostasine, Hoffman La Roche, Switzerland) was dissolved in physiological saline (100 IU/mL), and kept at –20°C until used. The fibrinolytic inhibitor tranexamic acid (trans-4-aminomethyl-cyclo-hexane-carboxylic acid, AMCA, Kabi Pharmacia, Stockholm, Sweden) was dissolved in physiological saline (80 mg/mL).

Thiobutabarbital (Inactin) was supplied by Sigma-Aldrich, Copenhagen, Denmark. The elastase inhibitor, ICI 200,355 [4-(4-bromophenylsulfonyl-carbamoyl)-benzoyl-L-valyl-L-proline 1(RS)-(1-trifluoroacetyl-2-methylpropyl)amide] was supplied by ICI Americas Inc., Wilmington, DE, USA. It is a substituted trifluoromethylketone with a molecular weight of 731.57 g/mol and an inhibition constant versus human leukocyte elastase of 0.5 nM.

Vectastain reagent was supplied by Vector laboratories Inc., Burlingham, CA, USA and Streptomyces hyaluronidase by Seikagaku Fine Biochemicals, Tokyo, Japan. Bovine serum albumin, iodoacetic acid, amino-n-caproic acid, benzamidine, and pepstatin A were supplied by Sigma Chemical Co., St Louis. MO, USA. Ethylene-diamine tetraacetic acid (EDTA) was supplied by Merck, Darmstadt, Germany and soy bean trypsin inhibitors by Worthington, Freehold, NJ, USA.

### Animal preparation

Rats were anesthetized intraperitoneally with 125 mg/kg of inactin, placed in a supine position and tracheostomized. A tracheal cannula (PE 240, Clay Adams, Becton Dickinson & Co., USA) was inserted for airway support, and an abdominal cannula (Portex; outer diameter 0.80 mm, Hythe, Kent, England) was inserted into the peritoneal cavity for AMCA injection and maintenance of anesthesia. All rats breathed spontaneously through the tracheostomy, and body temperature was maintained at 38°C with an electric pad. An intravenous injection site was prepared in both saphenous veins using polyethylene catheters (Portex; outer diameter 0.80 mm, Hythe, Kent, England) and 26-gauge needles to inject the elastase inhibitor and thrombin. The elastase inhibitor was dissolved in 4 mL of 0.1 M Na-phosphate buffer (pH 8.0) and diluted with phosphate-buffered saline (PBS) (40 μg/mL). The elastase inhibitor was then administered continuously via the saphenous vein by an infusion pump (B. Brown Apparatebau, Melsungen, Germany) for approximately 2 h at a rate of 200 μg/kg h^-1^.

A catheter (PE 50, Clay Adams) inserted into the right common carotid artery was advanced via the aortic arch into the left ventricle for injection of microspheres. Two other polyethylene catheters (PE 50, Clay Adams) were inserted and positioned into the right and left femoral arteries for pressure recordings and withdrawal of reference blood samples, respectively. The position of the catheter was confirmed by pressure tracing and post-mortem examination. A calibrated pump (model 355, Saga Instrument, New York, USA) was used for determination of liver blood flow and cardiac output.

**Figure 1.**
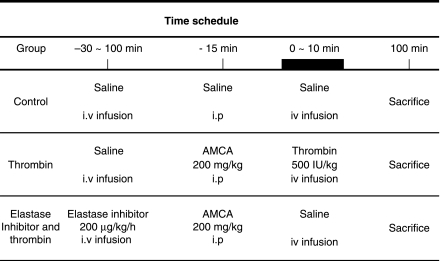
Experimental protocol.

Thrombin-induced lung injury was induced according to the experimental protocol (Figure 1) and as described previously (14). In brief, 15 min before thrombin infusion, rats were injected through the abdominal catheter with 200 mg/kg of AMCA to prolong fibrin entrapment in the lungs and increase pulmonary damage (19); 500 NIH units/kg of bovine thrombin were injected manually into the saphenous vein over a period of 10 min, using a stop-watch and a 1.0 mL disposable syringe.

Rats were killed 90 min after the end of the thrombin infusion. The abdomen was opened, and the aorta was gently freed to the level of the bifurcation of the descending aorta. Aortic blood samples were drawn into sodium citrate tubes from the aorta for determination of plasma HA, centrifuged at 400 × *g* for 10 min, and kept at –20°C until analyzed. The lungs were excised, cleaned gently with gauze, weighed for wet weight, and dried at 37°C in a warm incubator for approximately 72 hours. The lungs were reweighed for dry weight until their weight was constant.

### Wet weight to dry weight ratio and water content in the lung tissue

The wet weight to dry weight ratio (WW/DW) was determined in the left lung. The relative water content was also calculated in the left lung as described previously (5): the relative lung water content (%) = [( WW–DW ) / WW] × 100

### Bronchoalveolar lavage

Bronchoalveolar lavage was performed by a previously described method (20) with slight modifications. In brief, animals were killed by aortic exsanguination 90 min after termination of the thrombin infusion. The lungs were carefully dissected to the level of the carina after removal of the heart. The left main stem bronchus was tied proximally and then removed and used for determination of the WW/DW. The right lung was perfused *in situ* immediately after death by intratracheal infusion of five 3-mL aliquots of PBS at pH 7.4. Infusions were made under gravity at a constant hydrostatic pressure below 20 cmH_2_O. The lavage fluid was kept in the lungs for 3 min and recovered by gravity into 50 mL polystyrene tubes on ice. The recovery was 13.9 mL of total 15 mL. The lavage fluid was centrifuged at 400 × *g* for 10 min, and the supernatant was kept frozen at –20°C until analyzed.

### Extraction of lung tissue HA

The HA was extracted from the pulverized dried lung with 0.5 M NaCl. The HA was also under dissociative conditions, mixed with 4 M guanidinium chloride in 0.5 M sodium acetate buffer, pH 5.8, in order to extract HA possibly aggregated with proteoglycans. Twenty milligrams of the lung material were extracted with 2 mL of buffer for 16 hours under constant shaking at 4°C.

The samples were then centrifuged for 15 min at 2000 × *g*. The supernatants were recovered and the HA concentration determined. In order to destroy any proteoglycan or link protein that might have interfered with the HA assay, the guanidinium chloride extracts were treated with trypsin. The extract was diluted with 10 volumes of 0.1 M Tris/sodium acetate/HCl buffer, pH 7.3. Two units of pronase (Pronas 200 units/mL, Boehringer, Mannheim, Germany) were added, and the solution was incubated overnight at 37°C. The enzyme was then heat-inactivated at 100°C for 15 min. The HA concentration in a reference sample of HA (1.0 μg/mL) was not influenced by the enzymatic and heat treatment.

### Localization of HA in lung tissue

Staining for HA was performed by a previously described technique (10) on sections that had been fixed in cetylpyridinium chloride buffered formalin and embedded in paraffin; 3 μm sections were cut from the base of the lung to the apex and put on gelatin-coated slides, deparaffinized, and brought through a graded alcohol series to water. The sections were incubated first with 3% H_2_O_2_ for 5 min and then with bovine serum albumin, 10 mg/mL, for 30 min. After washing (2 × 10 min) with PBS, pH 7.3, sections were incubated overnight at 4°C with HA binding region from bovine nasal cartilage chondroitin sulfate proteoglycan core protein (HABP)-biotin (50 to 60 μg/mL). After two 10 min washes in PBS, sections were incubated with Vectastain Elite ABC Reagent for 30 min. After three further 10 min washes in PBS, they were then incubated for 5 min in ethylcarbazole-DMSO.

Control sections were incubated for 2 h in a humid chamber at 37°C with 500 unit/mL of Streptomyces hyaluronidase in 100 mM sodium acetate buffer, pH 5.8, in the presence of protease inhibitors, 1.8 mM EDTA, 1.8 mg/mL soy bean trypsin inhibitor, 2.0 mM iodoacetic acid, 0.18 mM amino-n-caproic acid, 9.0 mM benzamidine, and 1.8 μg/mL pepstatin A. Sections were mounted under glass covers in Kaiser’s glycerin-gelatin and examined by one observer without knowledge of the experimental conditions of the individual animals. Sections were then counterstained with Mayer’s hematoxylin. Photographs were taken using Normaski optics, on sections stained for HA alone, and bright-field microscopy was performed on sections counterstained with hematoxylin. The specificity of the reaction was checked by pretreatment of sections with Streptomyces hyaluronidase.

### Determination of HA

Concentrations of HA in plasma, BALF, and lung tissue extracts were determined in duplicate with a commercially available specific radiometric assay (HA-50-test, Pharmacia Diagnostics, Uppsala, Sweden), based on the use of specific HABP. HA from the samples or HA-standards (100 μL) was allowed to bind ^125^I-labeled HA binding region in solution (200 μL) for at least 60 min at 4–22°C. The unbound ^125^I-labeled HA binding region was then quantified after interaction with HA covalently bound to Sepharose particles. Particles were separated by centrifugation (1500 × *g*) after decanting the suspension. Radioactivity bound to the particles was then measured in a gamma counter. Sample concentrations were read from a standard curve after plotting percent radioactivity bound for standards against HA concentration on a line-log paper. The detection limit for HA was less than 5 μg/L, and variability of the measurements of HA was less than 10%.

### Determination of cardiac output and liver blood flow

Cardiac output and liver blood flow were measured as described earlier (21). In brief, microspheres with a diameter of 15 μm, labeled with ^57^Co, ^85^Sr, ^95^Nb, ^46^Sc, and ^65^Zn, were used. Each injection comprised approximately 0.4 × 10^6^ microspheres, suspended in 0.2 mL of homologous plasma. The microspheres were thoroughly mixed on a vibrator and immediately thereafter injected into the left ventricle during 15–20 s. An arterial reference sample was drawn at a rate of 0.49 mL/min using a calibrated pump, which was started immediately before the microsphere injection was begun and continued until 90 s after its commencement. For each microsphere injection, the type of isotope was chosen randomly. The choice of isotope did not influence blood flow data. Cardiac output and liver blood flow were calculated by means of a computer program (22).

### Statistical analyses

Statistical significances were tested by one-way analysis of variance amongst groups. A *t* test was used to determine the statistical significance of changes in HA concentration in BALF between groups. Data are expressed as means ± SEM.

## Results

### Biochemical variables

Administration of thrombin resulted in increased WW/DW ratios and relative lung water content (*P* < 0.001) (Table I). Thus, after infusion of thrombin, the WW/DW ratio increased by 42.3% and lung water content by 7.0%. Pretreatment with the elastase inhibitor caused a decrease in WW/DW and lung water contents (*P* < 0.001), which, however, remained significantly elevated compared to controls. The HA concentration in wet lung tissue decreased after thrombin infusion (*P* < 0.001). The elastase inhibitor counteracted this decrease (*P* < 0.05), but the levels of lung HA in this group remained lower than in the controls (*P* < 0.01). After thrombin treatment, the HA concentration in dried lung tissue was lower than in the control group (*P* < 0.001). The elastase inhibitor tended to inhibit this decrease, but the difference did not attain statistical significance. The HA concentration in plasma was about four times higher in the thrombin group than in the control group (*P* < 0.001). This increase diminished in the presence of the elastase inhibitor (*P* < 0.01). Thrombin treatment effected a moderate increase of the HA concentration in BALF (*P* < 0.05).

**Table I.** Effect of a leukocyte elastase inhibitor on wet weight to dry weight (WW/DW) ratio, relative lung water content, and concentration of HA in lung tissue and plasma and bronchoalveolar lavage fluid in rats with thrombin-induced pulmonary edema.

Measurements	Control	Thrombin	Thrombin and inhibitor
WW/DW ratio	5.07 ± 0.04 (13)	7.12 ± 0.13 (21)***	6.08 ± 0.29 (5)**^,†††^
Lung water content, %	80.3 ± 0.2 (13)	85.9 ± 0.3 (21)***	83.4 ± 0.8 (5)***^,†††^
Lung HAWW, μg/g WW	25.7 ± 1.3 (13)	14.0 ± 0.7 (21)***	18.9 ± 1.8 (5)**^,†^
Lung HADW, μg/g DW	130.3 ± 6.1 (13)	98.4 ± 4.1 (21)***	113.2 ± 8.6 (5)
Plasma HA, ng/mL	50.5 ± 3.6 (16)	212.5 ± 30.7 (15)***	79.5 ± 13.9 (7)*^,††^
BALF HA, ng/mL	16.5 ± 0.8 (8)	20.1 ± 1.1 (11)*	not determined

Data are expressed as means ± SEM. Number of rats in brackets.

*P < 0.05,

**P < 0.01,

***P < 0.001 versus control group.

^†^*P* < 0.05,

^††^*P* < 0.01,

^†††^*P* < 0.001 compared to the animals given thrombin only.

BALF = bronchoalveolar lavage fluid; DW = dry weight; HA = hyaluronan; HADW = lung HA concentration per g DW; HAWW = lung HA levels per g WW lung tissue; WW = wet weight.

### Histology

**Figure 2.**
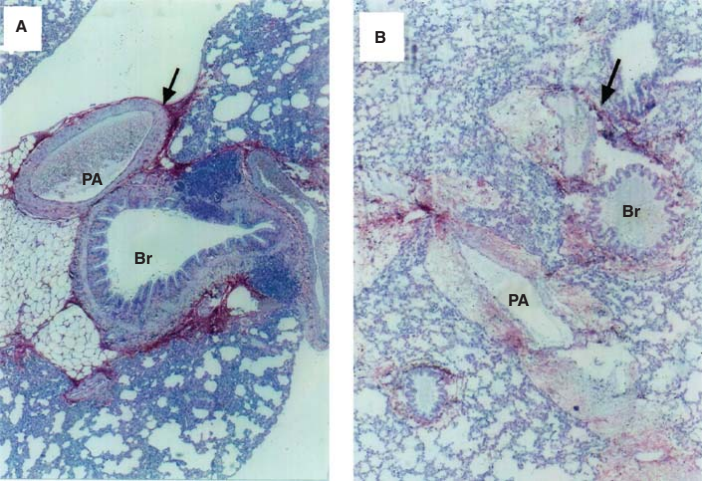
A: Staining of lung sections with biotin-avidin-hyaluronan-binding protein in a control rat. The red area represents hyaluronan-specific staining. The arrow indicates the intense staining of the perivascular and peribronchiolar space. The section was counterstained with hematoxylin (×140). B: Staining of lung sections with biotin-avidin-hyaluronan-binding protein in a representative rat with thrombin-induced pulmonary injury. The red area represents hyaluronan-specific staining. The arrow indicates the weaker staining of hyaluronan in the perivascular and peribronchiolar space. The section was counterstained with hematoxylin (×140). (PA = pulmonary artery; Br = bronchus.)

In control rats HA was found in the bronchial cartilage, submucosal tissue of bronchi and bronchioles, and the adventitia of arteries and veins. Slightly positive staining for HA was not only observed in the loose connective tissue surrounding bronchi and arteries, but also in the alveolar membranes (Figure 2A). In the thrombin-treated animals, HA staining in the lungs was markedly decreased and had changed into a fenestrated form, and HA in both the perivascular area and alveolar wall was weakly stained. HA had partly disappeared from the margin of vessels and cartilage, where the perivascular space was widened (Figure 2B).

### Cardiac output and liver blood flow

**Figure 3.**
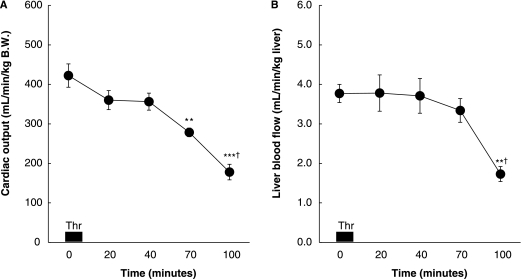
A: Cardiac output (CO; mL/min/kg B.W.) before (0) and 10, 30, 60, and 90 min after the end of thrombin (Thr) infusion in six rats with thrombin-induced pulmonary injury. Data are expressed as mean ± SEM. Baseline (0 min) represents steady-state values before thrombin infusion. ** *P* < 0.01 compared to the baseline values in CO. *** *P* < 0.001 compared to the baseline values in CO. † *P* < 0.05 compared to CO measured at 60 min after thrombin infusion. B: Liver blood flow (LBF; mL/min/kg liver) before (0) and 10, 30, 60, and 90 min after the end of thrombin (Thr) infusion in eight rats with thrombin-induced pulmonary injury. Data are expressed as mean ± SEM. Baseline (0 min) represents steady-state values before thrombin infusion. ** *P* < 0.01 compared to the baseline values in LBF. † *P* < 0.05 compared to LBF measured at 60 min after thrombin infusion.

Cardiac output was well maintained until 30 min after termination of the thrombin infusion and then progressively decreased (Figure 3). Sixty min after the infusion cardiac output was lower (*P* < 0.01) and was further decreased 90 min after termination of the thrombin infusion (*P* < 0.05). Liver blood flow was well maintained until 60 min after the end of the thrombin infusion, but was decreased at 90 min (*P* < 0.05), compared to the baseline value and the value at 60 min (Figure 3).

## Discussion

Administration of thrombin resulted in pulmonary edema, which was accompanied by a decrease in the concentration of HA in the lungs and an increase in HA in the plasma and bronchoalveolar lavage fluid, indicating increased wash-out of HA from the lung parenchyma. Histologically, staining for HA was decreased in the perivascular and peribronchiolar areas in thrombin-treated rats. To the best of our knowledge this is the first time that a decreased hyaluronan content in lung tissue has been described in a model of ARDS.

The decrease of HA in the lungs after administration of thrombin as determined both by biochemical and histological methods contrasts with findings in previous studies, where the concentration of lung HA was found to be increased in similar types of lung injury (7,8,10). One reason for this difference may be an increased synthesis of lung HA in their studies. Such an increase in lung HA synthesis should not occur in our acute experiments.

The metabolic degradation of HA is carried out by three acid hydrolases, hyaluronidase, β-N-acetylglucosaminidase, and β-glucuronidase (23). Rat alveolar macrophages are known to possess HA-degrading enzymes (24), which are activated by thrombin (25) and myeloperoxidase (26). In a previous study, myeloperoxidase was increased after thrombin infusion (27). Thus, it is conceivable that the activity of HA-degrading enzymes might be increased in the injured interstitium due to activation of alveolar macrophages, which leads to further degradation of HA in the lung tissue.

Polymorphonuclear leukocytes (15) and leukocyte elastase (14) are associated with the development of thrombin-induced lung injury. Toxic oxygen radicals (16) and myeloperoxidase (17) produced by thrombin-activated neutrophils (28) can directly degrade HA. Leukocyte elastase may cleave extracellular matrices such as proteoglycans, collagen, and fibrin that are bound to HA (18). Thus, HA bound to the extracellular matrix may be released and subject to oxygen-induced attack. This injury may lead to local degradation of HA, facilitating the wash-out of HA from the lung, and thus contributing to the increase in plasma HA.

In the present study, HA-stained microsections showed a decrease in HA in the perivascular and peribronchiolar areas in thrombin-treated rats. The perivascular and peribronchial location of lung HA (5) may indicate a stabilizing function of this molecule. Recent observations suggest that interstitial HA may protect against acute inflammatory changes in elastase-induced lung injury (29). Treatment with HMW-HA attenuated sepsis-induced lung injury in mechanically ventilated rats (30). These results indicate a low lung HA content after lung injury. If HA stabilizes lung microvessels and small airways, a reduction of HA may diminish the strength of the microvascular and bronchiolar barrier. Furthermore, HA acts as an osmotic buffer and may contribute to the water homeostasis in the interstitium. HA may also be of importance in the regulation of protein transport in the interstitium. If the lung HA concentration is decreased in the late phase of a thrombin-induced lung injury, water and protein might be extravasated from injured endothelial barriers into the alveolar space and pass without impedance through the interstitium, which may promote interstitial and alveolar edema.

Our results in this model of ARDS are consistent with the observations by Hällgren et al. in human ARDS (7) and by Berg et al. in a porcine model of injury and sepsis (31) that the HA concentration increased in BALF and blood. However, although these authors did not measure HA in lung tissue, they speculated that it was increased and contributed to pulmonary edema by immobilization of water. Somewhat in contrast, our findings suggest that lung HA counteracts the development of pulmonary edema and a shortage of this molecule in the lungs may contribute to the establishment of edema. This idea is strongly supported by the finding that administration of HMW-HA decreased the lung injury induced by sepsis in mechanically ventilated animals (30).

A decreased concentration of HA and its major transmembrane glycoprotein receptor, CD44, has been shown to increase unwanted human neutrophil elastase activity (32–34) and also to promote leukocyte extravasation and formation of pro-inflammatory cytokines (35,36). In the present study, the concentration of HA in plasma was increased 90 min after thrombin infusion, at which time liver blood flow and cardiac output were decreased. This is consistent with a recent observation in porcine peritonitis (37) and in patients with ARDS (1). The reduced hepatic blood flow is probably a result of the decreased systemic blood pressure (38) and cardiac output, leading to a reduced capacity for hepatic degradation of HA. A correlation between decreased hepatic blood flow and impaired liver function has been previously observed (39). Thus, one mechanism underlying the increased HA concentration in the plasma may be a decreased rate of clearance by liver endothelial cells due to a diminished liver blood flow.

To summarize, in this model of ARDS, the HA concentration in lung was decreased, and HA staining confirmed a reduction in HA, both in the perivascular and peribronchiolar areas. HA concentrations in BALF and plasma were elevated, indicating increased wash-out of HA from the lungs. A leukocyte elastase inhibitor counteracted not only the decrease in HA in lung tissue, but also the increase in plasma HA. The findings suggest that a decrease in HA in the lung may play a role in the pathogenesis of thrombin-induced lung edema, e.g. by reducing the stability of the microvascular and peribronchiolar barriers, and that leukocyte elastase is associated with the decrease in HA content in lung. An elevated plasma level of HA may serve as an indicator of the lung damage, at least in this ARDS model.
